# Proceedings of the Pennsylvania State Medical Society

**Published:** 1857-07

**Authors:** 


### PROCEEDINGS OF THE PENNSYLVANIA STATE MEDICAL SOCIETY.

THE Annual Convention of the Medical Society of the State of Pennsylvania assembled at Westchester on Wednesday, May 27th, 1857; Dr. R. La Roche, President, in the chair. The venerable Dr. Darlington welcomed the Society to Westchester, in a brief but very appropriate address. The following delegates were present:

*Beaver County*—Drs. David Minis, Jr., Oliver Cunningham.

*Berks County*—Drs. Martin Luther, P. G. Bertolet, Frank Rieser, Penrose Wiley.

*Blair County*—Dr. John Feay.

*Bradford County*—Dr. G. F. Horton.

*Carbon County*—Dr. G. B. Linderman.

*Chester County*—Drs. Isaac Thomas, W. Worthington, Isaac R. Walker, Elisha Gatchell, William H. Gunkle, W. W. Townsend, Benjamin Thompson.

*Lancaster County*—Drs. John L. Atlee, Sr., Adam Schiller, John Ream, P. Cassidy, T. Ellmaker, J. Augustus Ehler, Henry Carpenter, Isaac C. Weidler, J. D. Stubbs.

*Montgomery County*—Drs. William R. Ramsey, Hiram Corson, Milton Newberry.

*Perry County*—Dr. Benjamin P. Hooke.

*Philadelphia County*—Drs. R. La Roche, Wilson Jewell, Isaac Remington, N. L. Hatfield, Edwin Janvier, G. W. Norris, S. L. Hollingsworth, William Mayburry, Alfred Stillé, J. B. Biddle, R. H. Townsend, R. P. Thomas, Samuel Lewis, A. L. Kennedy, John Neill, J. Aitken Meigs, Edward Hartshorne, Ellwood Wilson, L. Curtis, D. D. Clark, George Martin, P. W. Russell, Henry Hartshorne.

*Schuylkill County*—Drs. F. J. Kern, J. S. Carpenter.

*Washington County*—Drs. Boyd Emery, George H. Cooke, Stephen L. Blachley.

Dr. La Roche, the President, delivered a very able and elaborate address, for which, on motion of Dr. Ehler, the thanks of the Society were tendered, and a copy was requested for publication in the Transactions.

At the afternoon session, in the absence ot the President, Dr. W. Worthington, Vice-President, occupied the chair.

On motion of Dr. Mayburry, Dr. John Curwen, of Harrisburg, was admitted as a delegate from Dauphin County. On motion of Dr. W. Jewell, Drs. T. G. Richardson and Percy La Roche, of Philadelphia, were invited to seats as members of the Convention.

Sanitary Reports were received from the Medical Societies of Philadelphia,

Perry, Beaver, Bradford, Montgomery, Blair, Schuylkill, Chester, and Washington Counties. After being read, either in substance or by abstract, the reports were referred to the Committee of Publication.

The Treasurer, Dr. R. P. Thomas, presented a report, which, after being audited, was pronounced correct, and adopted.

Dr. Atlee, on behalf of the Medical Profession of Lancaster, invited the Society to hold its next annual Convention in that city.

A report was received from Dr. John Bell, of Philadelphia, Chairman of the Committee, appointed in 1856, to memorialize the city authorities upon the importance of a more efficient system of public vaccination. On motion of Drs. Atlee and Mayburry, the subject of the report was referred to a Committee, consisting of one member from each County represented in this Convention; with authority to memorialize the State Legislature, in favor of the enactment of laws providing for general vaccination throughout the State.

The Committee appointed to address the Legislature in regard to printing the Transactions of the State Medical Society, was, by request, continued.

Dr. H. Carpenter, of Lancaster, read a report on the subject of vaccination and the vaccine virus; which was referred to the Committee of Publication. An interesting discussion ensued upon the subject of this report; in the course of which, Dr. Atlee, of Lancaster, related a case in which small-pox had occurred twice in the same child, under two years of age.

On motion of Dr. Carpenter, the appointment of Drs. Emerson and Condie, of Philadelphia, as a Committee to procure vaccine virus fresh from the cow, was renewed and continued.

Dr Biddle, of Philadelphia, on behalf of the Committee to which were referred the Resolutions of the Berks County Agricultural Society, in regard to the adulteration of drugs, reported that the subject had been taken in charge by the Pharmaceutical Convention; and moved, that the Committee be continued; which was agreed to.

The Committee appointed, last year, to confer, if desired, with officers of Insurance Companies, upon the subject of Vital Statistics, was continued.

The report of the Committee appointed to consider the propriety of issuing a Form of Record for cases of diseases, &c., and of passing resolutions tending to compel the preservation of sanitary statistics, was read, and, after modification, adopted. On motion of Dr. J. S. Carpenter, of Pottsville, the plan of a Form of Record, proposed by Dr. H. Hartshorne, last year, was referred to the Committee of Publication, with discretionary powers.

The report of the Committee on Biographical Notices of Deceased Members was received and referred to the Committee of Publication.

The preamble and resolutions proposed by Dr. R. P. Thomas, in 1856, in regard to a proposed alteration in the times of meeting of the American Medical Association, were taken up and discussed. On motion of Dr. Biddle, they were referred to the different County Medical Societies; with a request to report to this Society next year, on this subject, and on that of the expediency of modifying the basis of representation in the American Medical Association.

The Committee on the Organization of New County Medical Societies made a brief report.

Dr. Darlington invited the members of the Society to visit the Collection of specimens of Natural History in the Westchester Cabinet of Natural Science. The invitation was accepted. An invitation was also accepted to visit the Institution of Mr. Bolmar. On Wednesday evening, the delegates were handsomely entertained at the residences of Drs. Darlington, Thomas, and Worthington.

Thursday Morning, May 28.

Dr. La Roche, President, in the chair. Reports from several County Medical Societies (already named) were received, read, and referred.

Dr. R. P. Thomas presented a report from the Committee of Publication, which was adopted and ordered to be published.

On motion of Dr. Mayburry, Dr. Atlee was desired to communicate the facts which had occurred under his notice in regard to the late epidemic at Washington, D. C. An interesting discussion followed; in which Drs. Atlee, La Roche, Jewell, Corson, Remington, J. S. Carpenter, Neill, and others participated. By request of the Society, Hon. Mr. Hickman, member of Congress for the district, being present, gave a description of the disease as it occurred in his own case. The impression had been produced in his mind that the cause of the disorder was a mineral poison.

On motion of Dr. H. Hartshorne, the subject of the mode of extension of typhoid fever was recommended to the Epidemical Committees of the County Medical Societies, for special inquiry.

The following Resolutions, proposed by Dr. H. Carpenter, of Lancaster, were adopted.

*Resolved,* That the members of this Society be invited to present, at each meeting, prize essays, or original communications, which shall be subjects of discussion at such meetings.

*Resolved,* That the President, Vice-Presidents, Corresponding Secretary, and Treasurer, be constituted a Committee, to devise a plan for the extension of medical organization through the State; and to procure the services of some active and zealous member or members, who shall, during the ensuing year, visit the different counties in which no organization exists, call a sufficient number of the members of the profession together, explain to them the importance of organization, furnish them with the necessary documents, and adopt such other measures as may tend to serve the desired object; the expense thereof to be borne by this Society.

On motion of Dr. Jewell, Dr. S. D. Gross, of Philadelphia, was invited to a seat as a member of the Convention.

A report was received from the Board of Censors, relative to the Constitution and By-Laws of Delaware County Medical Society. On motion, subsequently, the Board was requested to hold the subject of that Society under consideration, and report again next year.

Dr. A. L. Kennedy read a report from the Committee on Forms of Sanitary Reports for County Medical Societies; recommending the introduction, as additional items, of information in regard to the medical effects of indigenous plants, and of new remedies; and, of facts in surgery and obstetrics. The recommendation was adopted.

Dr. Kennedy also moved that the Committee of Meteorology be requested to report to the Society on the means by which medical practitioners may most readily determine the electrical condition and fluctuations of the atmosphere; which was agreed to.

The Nominating Committee, appointed on Wednesday, reported a list of officers of the Society for 1858; who were, thereupon, elected.

Dr. John L. Atlee, of Lancaster, was chosen President. Vice-Presidents, Drs. J. B. Biddle, of Philadelphia; Isaac Thomas, of Westchester; Hiram Corson, of Montgomery County; Boyd Emery, of Washington County.

Recording Secretaries, Drs. Septimus A. Ogier, of Chester County, and Henry Hartshorne, of Philadelphia. Corresponding Secretary, Dr. Samuel Lewis, of Philadelphia. Treasurer, Dr. R. P. Thomas, of Philadelphia.

Committee of Publication, Drs. S. L. Hollingsworth, John Neill, and Edwin Janvier, of Philadelphia.

Censors were also appointed for the 1st, 2d, 3d, 4th, 5th, and 6th districts; and eleven gentlemen were selected to represent the Society in the American Medical Association.

After the announcement of his election, Dr. Atlee was conducted to the chair, and addressed the Society with much feeling. After the passage of resolutions of thanks, to the Chester County Delegation, to the Authorities of Westchester, to the Railroad Companies, and to the retiring officers, the Convention adjourned; to meet at Lancaster, on the last Wednesday in May, 1858.

On Thursday afternoon, the members enjoyed, by invitation, a delightful excursion to the banks of the Brandywine. In the evening, they were received by the members of the Chester County Medical Society, at a public entertainment, in Cabinet Hall. On the whole, although not numerously attended, this Convention has been one of considerable interest; and will leave agreeable recollections in the minds of all who were present.

